# Phylogeography of the Wheat Stem Sawfly, *Cephus cinctus* Norton (Hymenoptera: Cephidae): Implications for Pest Management

**DOI:** 10.1371/journal.pone.0168370

**Published:** 2016-12-13

**Authors:** Vincent Lesieur, Jean-François Martin, David K. Weaver, Kim A. Hoelmer, David R. Smith, Wendell L. Morrill, Nassera Kadiri, Frank B. Peairs, Darren M. Cockrell, Terri L. Randolph, Debra K. Waters, Marie-Claude Bon

**Affiliations:** 1 USDA ARS, European Biological Control Laboratory, 810, Avenue du Campus Agropolis, Montferrier sur Lez, France; 2 Montpellier-SupAgro, UMR CBGP, 755 avenue du Campus Agropolis, Montferrier sur Lez, France; 3 Department of Land Resources and Environmental Sciences, Montana State University, Bozeman, MT, United States of America; 4 USDA ARS, Beneficial Insects Introduction Research Unit, Newark, DE, United States of America; 5 Systematic Entomology Laboratory, USDA ARS, c/o National Museum of Natural History, Smithsonian Institution, Washington, D.C., United States of America; 6 Département Biologie-Ecologie-Environnement, Laboratoire de Zoogéographie, UMR 5175 CEFE, Université Paul-Valéry Montpellier 3, Route de Mende, Montpellier cedex 5, France; 7 Department of Bioagricultural Sciences and Pest Management, Colorado State University, Fort Collins, United States of America; 8 Northern Plains Agricultural Research Laboratory, USDA ARS, Sidney, MT, United States of America; National Cheng Kung University, TAIWAN

## Abstract

The wheat stem sawfly, *Cephus cinctus* Norton (Hymenoptera: Cephidae), is a key pest of wheat in the northern Great Plains of North America, and damage resulting from this species has recently expanded southward. Current pest management practices are inadequate and uncertainty regarding geographic origin, as well as limited data on population structure and dynamics across North America impede progress towards more informed management. We examined the genetic divergence between samples collected in North America and northeastern Asia, the assumed native range of *C*. *cinctus* using two mitochondrial regions (*COI* and *16S*). Subsequently, we characterized the structure of genetic diversity in the main wheat producing areas in North America using a combination of mtDNA marker and microsatellites in samples collected both in wheat fields and in grasses in wildlands. The strong genetic divergence observed between North American samples and Asian congeners, in particular the synonimized *C*. *hyalinatus*, did not support the hypothesis of a recent American colonization by *C*. *cinctus*. Furthermore, the relatively high genetic diversity both with mtDNA and microsatellite markers offered additional evidence in favor of the native American origin of this pest. The genetic diversity of North American populations is structured into three genetic clusters and these are highly correlated with geography. Regarding the recent southern outbreaks in North America, the results tend to exclude the hypothesis of recent movement of damaging wheat stem sawfly populations from the northern area. The shift in host plant use by local populations appears to be the most likely scenario. Finally, the significance of these findings is discussed in the context of pest management.

## Introduction

The wheat stem sawfly, *Cephus cinctus* Norton, (Hymenoptera: Cephidae) is one of the most serious pest for wheat production in North America with annual losses exceeding US$350 million [1]. The main damage to wheat is due to larvae developing within the stem where feeding damages translocating tissues and diminishes the photosynthetic capacity of the plant during the critical development period. Even more harmful, girdling of the stem by mature larvae weakens the ripened stems and causes lodging. Areas with historically high risk of damage due to the pest include the southern regions of the prairie provinces of Canada as well as the Northern Great Plains of the United States (*i*.*e*. Montana, North Dakota, South Dakota) [1, 2]. Recently, damage to winter wheat has expanded southward, especially into southeastern Wyoming, the Nebraska Panhandle, and, most recently, northeastern Colorado. Although *C*. *cinctus* was first observed in Colorado nearly 150 years [3], it was not until 2010 that damage was reported in cultivated winter wheat [4]. Tiller infestation levels up to 50% have been reported in different parts of Colorado [5].

Although the wheat stem sawfly is a key pest of wheat in North America, its origin remains obscure. This species was first reported and described from samples collected from wild grasses in Colorado in 1872 [3, 6]. It was first observed infesting wheat in Canada in Manitoba and Saskatchewan in 1895 [7]. Reports of *C*. *cinctus* infestations afterwards followed the westward movement of wheat production across the Canadian prairies and the northern US parts of the Great Plains [1]. *Cephus cinctus* has been considered for a long time as a North American species, originally attacking native grasses and gradually adapting to wheat (*i*.*e*. to spring wheat and more recently to winter wheat) after the introduction of the crop by European settlers in western North America. However, an alternative hypothesis, based on morphological observations from individuals of *Cephus hyalinatus* Konow collected in northeastern Asia emerged in the 1990's [8, 9]. The authors synonymized the Asian species *C*. *hyalinatus* under *C*. *cinctus*. They further suggested that several other Asian species, including *C*. *camtschatcalis* Enslin and *C*. *zahaikevitschi* Ermolenko, may also be conspecific with *C*. *cinctus*. Moreover, only four other species in the grass-mining tribe Cephini are present in North America. The European wheat stem sawfly, *Cephus pygmaeus* (L.) and the black grain stem sawfly, *Trachelus tabidus* (F.) are known to have been introduced from Europe [10, 11] and *Calameuta clavata* (Norton) and *Calameuta middlekauffi* Smith and Schiff which are poorly known species found in the western United States [12, 13]. Therefore, the native American origin has been questioned and the North American populations of the wheat stem sawfly may have been due to introduction from northeastern Asia during recent historical times [9]. Taken as a whole, the lack of conclusive evidence about its origin has impacted the effective management of this pest. In this study, we investigate the genetic divergence between the American *C*. *cinctus* and the synonimized *C*. *hyalinatus* collected in Asia, using two mitochondrial regions (*COI* and *16S*). These findings should provide new insights into the origin of North American wheat stem sawfly populations.

Current management of *C*. *cinctus* populations relies on negligible use of insecticides, host plant resistance programs and the optimization of conservation biological control and various tillage operations [1, 2]. However, traditional pest management practices are insufficient [1]. The patterns of genetic structure in natural populations may offer new insights into the evolutionary processes and the dynamics of natural populations, and such information can be further utilized for pest management. Genetic approaches have demonstrated their usefulness for insect pest management by allowing the reconstruction of invasion routes [14–16], the identification of cryptic species and divergent lineages [16, 17] and also estimating gene flow between populations [18, 19] and providing inference of past histories (*i*.*e*. population size changes, range expansions or contractions) [20]. Little is known about the population genetics of this pest, the data being restricted to a single study using RAPD markers to decipher distinct genetic units from Montana and North Dakota [21]. The authors also found that these genetically discrete entities exhibited different durations of post-diapause development. However, there is no genetic data available to date regarding either the recent southern outbreaks on wheat nor for North American populations of *C*. *cinctus* on wildland grasses, and understanding gene flow among those populations would provide valuable information. In the second part of the study, we characterize the structure of genetic diversity in the populations of the wheat stem sawfly in the main wheat producing areas of western North America using a combination of mtDNA marker and five microsatellites on samples collected both from wheat and from grasses in wildlands. Finally, the significance of these findings is discussed in the context of pest management.

## Material & Methods

### Ethics statement

*Cephus cinctus* and, more broadly members of the genus *Cephus*, are widely regarded as important insect pests of wheat. Research on these species is welcomed by farmers because understanding their biology and behavior may be useful to protect crops from losses due to these pests. Thus, for sampling carried out on private lands, we had permission from the owners. Collection of these species on public lands was conducted in compliance with existing regulations for insects defined as non-commercial, as determined by local offices. Additionally, these field studies did not involve endangered or protected species.

### Insect sampling and DNA extraction

Samples of *C*. *cinctus* were collected between 2001 and 2014 across two Canadian provinces and six US states (S1 Table). Sampling covered an extensive part of the species’ distribution on wheat, while sampling on wildland grasses was restricted to Montana and limited to areas predominated by native and invasive *Bromus* species, including one native and one invasive subspecies of *Bromus inermis* Leyss. (Poales: Poaceae) and native *Bromus marginatus* Nees ex Steud. (Poales: Poaceae) (S1 Table). Because of the haplo-diploid life cycle of *C*. *cinctus* [22], in which males develop from unfertilized eggs and are haploids, sex determination was required and only adults were used. Both males and females were used for mtDNA-based analyses but only females were kept for microsatellite analyses. All samples were stored in 96% ethanol at -20°C. Total genomic DNA was extracted from the entire insect (for most samples) and more recently from the head only.

Voucher specimens of *C*. *hyalinatus* were kindly provided by the National Museum of Natural History, Smithsonian Institution (Washington, D.C.) and comprised dried, pinned museum samples collected between 1960 and 1970 in Siberia, except for one specimen that was collected in Inner Mongolia (China) in 2002 and was preserved in alcohol (S1 Table). Additional fresh samples of *C*. *fumipennis*, a congeneric Chinese wheat pest [23, 24], were used as well as individuals of *C*. *pygmaeus* (S1 Table). After DNA extraction using a non-invasive method, voucher specimens were shipped back to the National Museum of Natural History, Smithsonian Institution in Washington, D.C. For the others, DNAs and specimens are held, respectively frozen and dried in collections of the USDA European Biological Control Laboratory (EBCL), Montferrier sur Lez, France.

Specimens of *C*. *hyalinatus* and *C*. *pygmaeus* were identified by Dr. David Smith (United States Department of Agriculture Systematic Entomology Laboratory, Washington D. C—USA). Specimens of *C*. *fumipennis* were identified by Dr. Aiping Liu (Institute of Grassland Research, Chinese Academy of Agricultural Sciences, Inner Mongolia—China) and subsequently confirmed by D. Smith.

For fresh samples, extraction of total genomic DNA was performed using Qiagen DNeasy Blood & Tissue kit following manufacturer’s protocol (July 2006) after several washes to remove ethanol. For museum samples, a non-destructive DNA extraction was performed using the DNeasy extraction protocol (Qiagen Inc) as modified by C.D. Zhu & J.S. Noyes (unpublished).

### Insights on the Origin of the Wheat Stem Sawfly

For this phylogenetic study, we focused on seven specimens of *C*. *cinctus*, collected in Canada, Montana and Colorado, these samples corresponding to the most common haplotypes of the two lineages found across North America (see section on Phylogeography of North American Wheat Stem Sawflies).

Polymerase chain reaction (PCR) amplifications were conducted for two mitochondrial gene fragments: a 264-bp region of the cytochrome c oxidase subunit I (*COI*) and a 442 bp of the ribosomal 16S RNA (*16S*). Additional details on primers and PCR conditions can be found in S1 Appendix. The purified PCR products were sequenced in both directions by Genoscreen (Lille, France) using an ABI PRISM 377 DNA sequencer. Sequences were edited in CodonCode Aligner (www.codoncode.com) and multiple alignments were performed using CLUSTAL W [25] as implemented in CodonCode. The constructed *16S* and *COI* sequence datasets were merged with the homologous regions obtained from the complete mitochondrion sequencing of four Cephidae species that were obtained from NCBI (*Calameuta idolon* Genbank accession number: KT260168; *Calameuta filiformis* KT260167; *Cephus pygmaeus* KM377623 and *Cephus sareptanus* KM377624).

To estimate divergence between each taxon, we used Mega version 7.0 [26] to calculate the average and pairwise genetic *p*-distances within and among species on single gene data sets.

Bayesian analysis was conducted using MrBayes 3.1.2 [27]. First, jModelTest2 [28] was used to test for the best-fit model of sequence evolution for each gene. The best-fit model was selected using the corrected Akaike Information Criterion (AICc) [28]. Two simultaneous runs of 3 million generations were performed and convergence was maximized by ensuring that the average standard deviation of split frequencies fell below 0.01 and potential scale reduction factors approached 1.0. The first 25% of each run was discarded as burn-in phase for the estimation of the consensus topology and the computation of the posterior probability for each node.

The Poisson tree processes (PTP) model for species delimitation [29] was used to identify the most likely species number in Bayesian phylogeny of the combined dataset. This model estimates the speciation rate directly from the number of substitutions and does not require ultrametric trees as inputs. This method hence assumes that each substitution has a small probability of generating a speciation event. Consequently, the number of substitutions between species is expected to be significantly higher than within species. The analysis was conducted on the web server for PTP (available at http://species.h-its.org/ptp/) with 200,000 MCMC generations, a thinning value of 100 and a burn-in of 25%. As recommended by the developers, the convergence of the MCMC chain was confirmed visually [29].

### Phylogeography of North American Wheat Stem Sawflies

#### Molecular analyses of mitochondrial DNA

The *COI* mitochondrial region was partially amplified (762 bp) with C1-J-2183 and TL2-N-3014 pair of primers as described by Simon et al [30] from 1 to 21 individuals per population. Among the 349 specimens, 270 were collected on wheat and 79 on wildland grasses. Additional details on samples and PCR reactions are provided in S2 Table and S1 Appendix respectively. All sequences were obtained in both forward and reverse senses, assembled into consensus contigs using CodonCode Aligner and then aligned using CLUSTAL W.

Intraspecific phylogenetic relationships were analyzed in two different ways. First, we reconstructed by Bayesian analysis using MrBayes 3.1.2. The methodology was the same as previously described. An haplotype network was then constructed in PopART [31] using TCS network (95% connection limit).

For further analysis, sampling locations containing one or two individuals were pooled with the nearest sampling site or excluded when the sites were too much isolated (> 30km). The estimation of gene diversity (*H*_*d*_) and nucleotide diversity (π) for each of the sampling locality was conducted with Arlequin 3.5 [32]. Demographic history changes were analyzed using two neutrality tests: Tajima’s *D* [33] and Fu’s *F*s [34]. These two frequency-based indicators of a population expansion (or selection in non-neutral markers) were calculated with Arlequin 3.5.

A spatial analysis of molecular variance (SAMOVA) was used to investigate geographical structure with SAMOVA 1.0 [35]. This approach defines groups of populations that are geographically homogeneous and maximally differentiated. The program was run for two to ten differentiated groups (*K* = 2 to *K* = 10) using 10,000 permutations from 100 random initial conditions. Each group defined by SAMOVA was analyzed separately for its gene diversity (*H*_*d*_) and nucleotide diversity (π). Allelic richness *r* was computed using the rarefaction method proposed by Petit et al [36] with Contrib (http://www.pierroton.inra.fr/genetics/labo/Software/Contrib).

Finally, a hierarchic analysis of molecular variance (AMOVA) was applied between collections from wildland grasses and wheat fields to test for host plant effect on the genetic structure of populations. This analysis was conducted with the software Arlequin 3.5.

#### Molecular analyses of microsatellite markers

Five microsatellite markers, described by Hartel et al [37] were used to genotype 539 individuals from 36 sampling sites (S2 Table). Additional details for genotyping protocols can be found in S1 Appendix.

Deviation from Hardy-Weinberg equilibrium and linkage disequilibrium between pairs of loci were tested with Genepop 4.2.1 [38]. Allelic richness (*AR*) with the rarefaction method, observed and expected heterozygosity (*Ho* and *He*) and inbreeding coefficients (*F*_is_) were estimated using FSTAT 2.9.3.2 [39].

To explore the population structure within the whole dataset, we used the Bayesian clustering approach implemented in Structure 2.3.4 [40]. An admixture model with correlated allele frequencies was used and simulations were run with sampling location as prior because in situations of low levels of genetic divergence or a limited number of loci, this model allows a more accurate detection of genetic structure [41]. The burn-in period of each run was set to 100,000 followed by 100,000 MCMC iterations. We performed 10 independent runs for each value of *K* ranging from 1 to 8. We assessed the uppermost level of population structure by using the Δ*K* method [42] implemented in Structure Harvester [43]. The graphical display of genetic structure was produced with Distruct [44].

Traditional methods may be useful for describing population structure and complementary to Bayesian methods [45], we also used as alternative methods a Principal Coordinates Analysis (PCoA) calculated via covariance matrix on standardized data in GenAlEx v 6.5 [46] and we built a population-based neighbor-joining (NJ) tree. The pairwise Cavalli-Sforza and Edwards’ chord distance measures [47] were calculated in Population 1.2.32 software [48] and the resulting distance matrix was used to build the NJ tree. The robustness of the nodes was evaluated by carrying out 1000 bootstrap replicates over loci. The NJ tree was visualized with Figtree v 1.4.2 [49].

The level of genetic differentiation among populations was quantified by the estimation of the pairwise *F*_ST_. Estimation of *F*_ST_ values and their statistical significance was conducted using Arlequin 3.5. Isolation by distance (IBD) was examined within the whole dataset and separately for the groups detected with Structure. IBD was investigated by testing the correlation between pairwise *F*_ST_ and geographical distances using the Mantel test as implemented in Genepop.

## Results

### Insights on the Origin of the Wheat Stem Sawfly

The two loci (*16S* and *COI*) were successfully amplified from all specimens. All haplotypes discovered in this study were deposited in GenBank under the accession numbers reported in S1 Table. We aligned the resulting sequences with sequences obtained from complete sequencing of the mitochondrial genome of four species published by Korkmaz et al [50, 51]. Consequently, the *COI* sequences were aligned with a 264 bp portion and the *16S* sequences were aligned with a 442 bp portion.

**Fig 1:**
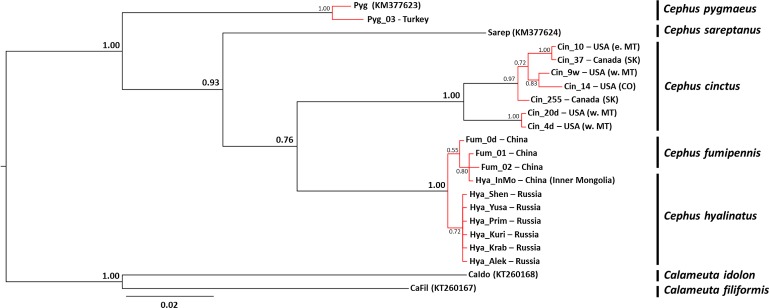
Bayesian phylogenetic tree from analysis of the combined mtDNA dataset. Posterior probabilities associated with major nodes are indicated in bold. Branch lengths represent expected substitutions per site. The scale bar indicates the expected number of substitutions per site. Results of the PTP analysis are provided using colored branches. Monophyletic groups in red indicate a single putative species as well as terminal branches in black. Names of terminals indicate codes of the samples and the GenBank number follows the names when sequences were obtained from Genbank. Names in bold after a | symbol are taxonomic or geographic identifiers of the putative species.

The most appropriate model of *COI* sequence evolution was HKY+G while it corresponded to the GTR+I model for the *16S* dataset. The Bayesian phylogenetic tree based on the combination of the two mtDNA markers was well supported as major nodes were supported by posterior probabilities ≥ 0.76 (Fig 1). The PTP model identified a total of seven putative species (Fig 1) within the dataset with most results being congruent with morphological identification. Surprisingly enough, *C*. *fumipennis* and *C*. *hyalinatus* clustered together as a single molecular species. Other results were congruent with morphological identification. The divergence was very low between the Asian species (*i*.*e*. *C*. *fumipennis* and *Cephus hyalinatus*) in the *COI* dataset and null in the *16S* dataset (Table 1).

**Table 1:** Comparison for each gene (above diagonal: *16S* and below diagonal: *COI*) of the average pairwise divergences (*p*-distance, expressed as a %) between the different groups used in the study.

	*Cephus cinctus*		*Cephus fumipennis*	*Cephus hyalinatus*	*Cephus pygmaeus*	*Cephus sareptanus*	*Calameuta idolon*	*Calameuta filiformis*
	Lineage 2	Lineage 1						
*Cephus cinctu*s—Lineage 2		2.3 (±0.7)	5.3 (±1.0)	5.0 (±1.0)	5.8 (±1.2)	5.6 (±1.2)	9.3 (±1.3)	8.4 (±1.3)
*Cephus cinctu*s—Lineage 1	2.5 (±0.9)		5.0 (±1.0)	5.3 (±1.0)	6.6 (±1.0)	6.0 (±1.1)	9.0 (±1.3)	8.2 (±1.3)
*Cephus fumipennis*	9.2 (±1.6)	7.7 (±1.7)		0.0 (±0.0)	5.7 (±1.1)	5.5 (±1.0)	9.0 (±1.3)	8.5 (±1.3)
*Cephus hyalinatus*	9.5 (±1.6)	8.0 (±1.7)	0.10 (±0.5)		5.7 (±1.1)	5.5 (±1.0)	9.0 (±1.3)	8.5 (±1.3)
*Cephus pygmaeus*	10.3 (±1.8)	11.1 (±1.7)	11.7 (±1.8)	11.9 (±1.9)		6.6 (±1.2)	8.4 (±1.3)	8.1 (±1.3)
*Cephus sareptanus*	9.7 (±1.8)	10.7 (±1.8)	12.6 (±1.9)	12.6 (±1.9)	12.3 (±1.9)		8.2 (±1.3)	9.0 (±1.4)
*Calameuta idolon*	14.6 (±2.1)	14.6 (±2.1)	13.4 (±2.0)	13.4 (±2.0)	12.1 (±1.9)	13.4 (±2.0)		7.6 (±1.2)
*Calameuta filiformis*	15.6 (±2.2)	15.7 (±2.2)	15.8 (±2.2)	16.0 (±2.2)	15.3 (±2.1)	11.9 (±2.1)	10.0 (±1.8)	

There were two lineages of *C*. *cinctus* that were also identified as two different species by the PTP model. The pairwise divergences observed between the Lineage 1 and 2 (2.5% (±1.6) and 2.3% (±0.7) for *COI* and *16S* respectively) were lower than the pairwise divergences observed at the congeneric level (Table 1). However, it was five times higher than the average intraspecific level for *COI* (0.5% ± 0.2) and ten times higher for *16S* (0.2% ± 0.1). The level of *COI* pairwise divergence between *C*. *cinctus* and *C*. *hyalinatus* was 8.0% (± 1.7) and 9.5% (± 1.6) for Lineage 1 and Lineage 2 of *C*. *cinctus*, respectively. The *16S* pairwise divergence was 5.3% (± 1.0) between *C*. *cinctus* and *C*. *hyalinatus* and 5.0% (± 1.0) for the Lineage 1 and Lineage 2 of *C*. *cinctus* (Table 1).

### Phylogeography of North American Wheat Stem Sawflies

**Fig 2:**
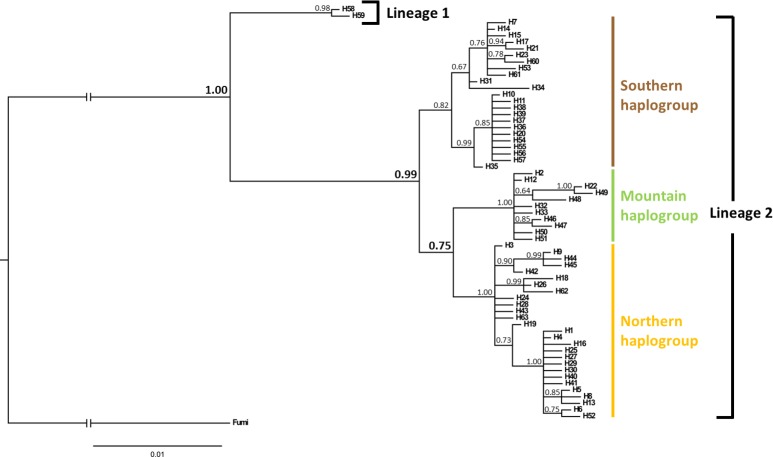
Bayesian phylogenetic tree from analysis of the *COI* sequences of North American *Cephus cinctus*. Posterior probabilities associated with major nodes are indicated in bold. Branch lengths represent expected substitutions per site. The scale bar indicates the expected number of substitutions per site. *Cephus fumipennis* was used as outgroup.

**Fig 3:**
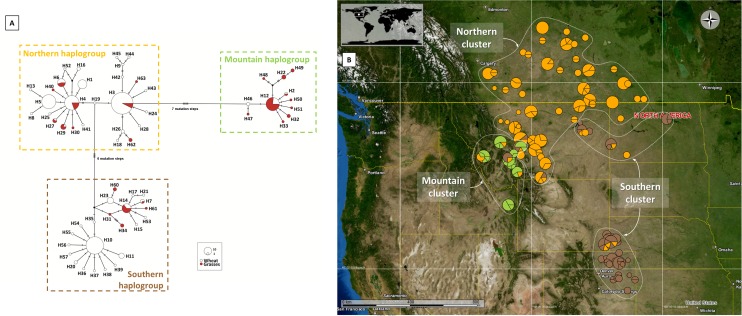
Phylogenetic relationships and genetic clustering *Cephus cinctus* across North America using a mitochondrial marker (*COI*). A. *COI* mitochondrial network of the North American *Cephus cinctus*. Each circle corresponds to one haplotype; circle size gives the proportion of individuals belonging to the haplotype. The color inside each circle represents the host and indicates the proportion of individuals sampled in the different hosts. Each link between circles indicates one mutational event. Black circles represent missing intermediate haplotypes. B. Geographic locations of *Cephus cinctus* samples used in the mtDNA phylogeography and distribution of *COI* haplogroups. Pie chart sizes are proportional to sample size and each haplogroup is colored according to the results of Bayesian tree and haplotype network. Dotted white lines represent the three groups identified by the SAMOVA.

*Mitochondrial DNA*. A fragment of the *COI* mtDNA gene from 349 individuals was amplified and sequenced. The final alignment comprised 762 bp and allowed the detection of 63 haplotypes. All sequences were deposited in GenBank (accession numbers: KX880513-KX880575). The Bayesian phylogenetic tree was well supported as major nodes were supported by posterior probabilities ≥ 0.75 (Fig 2). The topology displayed the presence of two divergent lineages and confirmed the relatively high divergence observed between the two *C*. *cinctus* lineages in the phylogenetic analysis. Lineage 1 was composed of two specimens collected in western Montana and Lineage 2 corresponded to all other sequences. Lineage 1 was not used in the subsequent analysis due to very low occurrence and putative status as a distinct species as detected in the PTP analysis. Lineage 2 of *C*. *cinctus* was split in three different mtDNA haplogroups (Fig 3). The three major mtDNA haplogroups resolved by the haplotype network consisted of one to three haplotypes at high frequency, with all other haplotypes radiating from these by a few substitutions (Fig 3A). The haplotypes in the first haplogroup were mainly distributed in Canada and eastern Montana (Fig 3B) whereas the haplotypes of the second haplogroup were strictly distributed across the Rocky Mountains (western Montana and Idaho). The third haplogroup was essentially found in populations from eastern Montana, North Dakota, and the southern populations from Wyoming, Nebraska and Colorado. Sixteen haplotypes were found only on grasses, nine were shared by grasses and wheat, and the rest was collected from wheat only (Fig 3A).

The largest increase in *F*_CT_ values was observed when the geographic sampling area was partitioned into three clusters (S1 Fig). The overall regional genetic groups delineated by the SAMOVA were: (i) the « Northern » cluster, largely distributed from Southern Canada to Montana, (ii) the « Mountain » cluster distributed across the Rocky Mountains from western Montana to Idaho and (iii) the « Southern » cluster composed of individuals from eastern Montana, North Dakota, Wyoming, Nebraska and Colorado (Fig 3B).

The total gene diversity was 1.000 (± 0.003) while nucleotide diversity was 0.012 (± 0.006). Reduced haplotype diversity and nucleotide diversity were observed in several populations distributed across the sampling area, mainly in Saskatchewan, Canada and Colorado, USA (S2 Table). When considering the clusters identified by the SAMOVA, both haplotype diversity and nucleotide diversity were highest in the Mountain cluster (Table 2), however using the rarefaction method, the allelic richness was the highest in the Southern cluster.

**Table 2:** Indices of genetic diversity per clusters identified in SAMOVA, Tajima’s *D* and Fu’s *Fs* statistics.

Clusters	*n*	H	Hd	± SD	π	± SD	*r*	Tajima's *D*	*p*	Fu's *FS*	*p*
Northern Cluster	156	19	0.774	0.024	0.004	0.002	11.681	-0.901	0.184	-4.224	0.090
Mountain Cluster	75	20	0.813	0.039	0.010	0.005	19.000	-0.939	0.188	0.518	0.518
Southern Cluster	90	28	0.687	0.056	0.005	0.003	23.395	-1.732	0.016	-13.730	0.001

*n*: Number of individuals analyzed; H: Number of haplotypes; Hd: Gene diversity and its standard deviation; *r*: Allelic richness after rarefaction; π: Nucleotide diversity and its standard deviation; Tajima's *D* and Fu's *FS*: results of neutrality test and their respective *p*–values.

Few Tajima’s *D* tests (for two sites in Montana, one site in Wyoming and one site in Colorado) and Fu’s *FS* tests (for one site in Colorado) showed significant negative values (S2 Table). When neutrality tests were applied for the different geographic groups identified in the SAMOVA, both Tajima’s *D* and Fu’s *FS* tests showed negative values and were significant for the Southern cluster, suggesting that this population had experienced a recent rapid expansion (Table 2). The tests were not significant for the two other clusters.

AMOVA showed that the genetic variation was quite evenly distributed among populations within groups (52.60%: *P* < 0.001) and within populations (40.75%: *P* < 0.001). Only a small and non-significant fraction of differentiation was attributed to host plants (6.65%: *P* = 0.060).

#### Microsatellites

No evidence for linkage disequilibrium within any of the five loci was observed. All loci showed polymorphism both within and among populations. Allelic richness was high (3.952 ≤ Ar ≤ 7.513), with highest values observed in the southern populations and the lowest in western Montana. Expected heterozygosity ranged from 0.512 to 0.770 (S2 Table), and the highest values were observed in the southern populations while the lowest ones were found in western Montana again. Significant heterozygote deficiency was observed in six populations mainly distributed in western Montana, except for one in Colorado. A positive *F*_is_ was observed in all of these populations and values ranged from 0.115 to 0.628.

**Fig 4:**
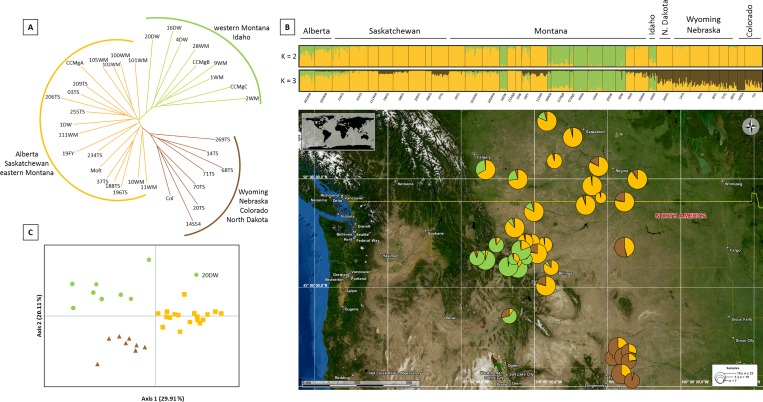
Genetic clustering of *Cephus cinctus* across North America using 5 microsatellites. A. Neighbor-joining tree based on the chord distances of Cavalli-Sforza & Edwards (1967) computed between the sampled populations. B. Above. Graphical representation of population genetic structure estimated by the Bayesian clustering approach implemented in Structure software. Provinces / states are indicated above the plots whereas sampling localities below. Each individual is represented by a vertical line and the proportion of each color corresponds to the percentage of coancestry in each genetic cluster. B. Below. Map of collections in the North America. Each location is represented by a pie chart showing ancestry (*Q*) among three genetic clusters as determined by analysis with Structure. Pie chart sizes are proportional to sample size. C. Principal coordinate analysis (PCoA) plot based on the genetic distance among the different populations. Each population is colored according to the Structure clustering (*K* = 3).

The NJ tree constructed from Cavalli-Sforza and Edward’s chord distances showed a split between three groups matching the clusters identified with Structure: (i) the populations from western Montana, (ii) the populations from the North (Canada and eastern and Central Montana) and (iii) all southern populations (Colorado, Wyoming, Nebraska) and the population from North Dakota (Fig 4A).

Results of the Structure clustering suggested a number of clusters of *K* = 2 in all runs, largely corresponding to western Montana *vs*. all other populations (Fig 4B). However, increasing the number of clusters to *K* = 3, the analysis suggested that populations from Colorado, Wyoming and Nebraska formed a distinct cluster corresponding to the Southern cluster. The population from North Dakota showed signs of admixture between this Southern cluster and the Northern one, the proportion of membership (*Q*) being *Q* = 53.4% for the Southern cluster and *Q* = 45.6% for the Northern cluster (Fig 4B). The principal coordinate analysis (PCoA) largely confirmed the Structure results and revealed the existence of three clusters (Fig 4C). These results are all congruent together and with the Structure analysis, except for one population (20DW) that was not grouped with the Mountain cluster as suggested by Structure.

The level of genetic differentiation among populations was low to moderate with pairwise *F*_ST_ values ranging from 0.001 to 0.221. Significant genetic differentiation among populations was observed and mostly non-significant *F*_ST_ values were found within clusters (S3 Table). The pairwise genetic differentiation between populations of the Mountain cluster and populations of the two other clusters was larger (Mountain-Northern: mean *F*_ST_ = 0.082 and Mountain-Southern: mean *F*_ST_ = 0.079) than the differentiation observed between populations of the Northern and Southern clusters (mean *F*_ST_ = 0.060).

Across all populations, the Mantel test of geographic *versus* genetic distances was statistically significant (*p* < 0.001) indicating a weak IBD, with a Mantel correlation coefficient (Mantel *r*) of 0.09, however, Mantel tests for IBD within each cluster were non-significant.

## Discussion

### Insights on the Origin of the Wheat Stem Sawfly

To date, only one study has examined the assumption of Asian native status of *Cephus cinctus* with morphological observations combined with historical data [9]. The present study is the first attempt at dealing with the origin of this pest using molecular data. Although the limited number of Asian specimens used here precludes concluding definitely about the origin, our findings support the hypothesis of a North American origin of *C*. *cinctus*. The genetic divergence for *COI* between *C*. *cinctus* and *C*. *hyalinatus* collected from the presumed native range of *C*. *cinctus* (*i*.*e*. Asia) and more specifically Siberia (*i*.*e*. the area where specimens of *C*. *hyalinatus* were synonymized as *C*. *cinctus* [8]) was within the range of the congeneric level observed in this study but also within the range reported in a previous work on other Cephidae [52]. This suggests that *C*. *cinctus* and *C*. *hyalinatus* correspond to two distinct species. This was further confirmed by the analysis of *16S* sequences [53] and the PTP analysis. Although the PTP procedure is not error-free and the resulting species delimitation is putative [29], this method has been implemented in several recent studies [17, 54–57] and the procedure appears to be quite robust to different phylogenetic methods [58]. Furthermore, the high genetic diversity observed across North America both on mtDNA and microsatellite markers is also compatible with the idea of a native area on this continent [59, 60]. Likewise, indirect line of evidence for a North Amercian origin comes from the literature. Efforts to locate foreign natural enemies for biological control of this pest were conducted in Asia and the parasitoid *Collyria catoptron* Wahl (Hymenoptera: Ichneumonidae), obtained from *C*. *fumipennis* was considered as a good candidate [23]. However, complete development was never observed in *C*. *cinctus*, indicating that it is not a suitable host for the parasitoid [61]. In the light of the genetic results presented here, this could be interpreted as another piece of evidence for a North American origin of the wheat stem sawfly. Although obtaining voucher specimens from Asia was challenging, a wider sampling of *C*. *hyalinatus* in northeastern Asia, combined with the genetic methods applied here, would allow to confirm or infirm the present results and definitively would help in elucidating the origin of this pest.

The present data confirm that a revision of the genus is clearly needed to understand species delineation. To our knowledge, there is no complete phylogeny of the *Cephus* genus. The only previous phylogeny of the Cephinae, based on another *COI* region, did not include either *C*. *cinctus* or *C*. *hyalinatus* or other potential Asian conspecifics [62]. Color differences are common criteria to distinguish between *Cephus* species, but coloration varies, hence it may cloud the systematics for those species [63]. An integrative taxonomic approach, using behavior, ecology, genetics and morphology, would be particularly relevant when dealing with morphologically closely related species complexes [17, 64].

### Phylogeography of North American Wheat Stem Sawflies

Among the 63 mitochondrial haplotypes, we observed two divergent lineages, Lineage 1 and 2. Lineage 1 corresponds to only two individuals collected in the Rocky Mountains in western Montana. Moreover, the divergence observed between the Lineage 1 and *C*. *hyalinatus* is high both for the *COI* and the *16S* sequences, suggesting that Lineage 1 corresponds neither to *C*. *hyalinatus* nor to any of the species considered. The evidence of two divergent lineages was unexpected as only two *Cephus* species are known from the USA [9, 23]. This result suggests two distinct genetic entities within the USA for which the systematic status as yet to be examined. The very low occurrence of the Lineage 1 and its narrow distribution restricted to the Rocky Mountains suggest that this lineage is not responsible for the wheat stem sawfly problem encountered by the North American wheat producers.

Lineage 2 can be split into three major haplogroups. The distribution of these haplogroups strongly suggests a geographic pattern which was confirmed by SAMOVA. Microsatellite results further supported the definition of the three clusters. The Δ*K* method suggested that the uppermost level of population structure was *K* = 2, however the Δ*K* method may underestimate the number of population clusters [45]. Therefore when increasing the number of clusters to *K* = 3, the resulting clustering corresponds largely to those observed with the mtDNA analysis. Moreover, the NJ tree provided similar qualitative outcomes and the PCoA was largely congruent with the definition of three clusters. Consequently, both mitochondrial and microsatellite data identified three genetic clusters in our sampling set (Figs 2–4). The « Northern » one is widely distributed among Canada as well as Central and eastern Montana. The « Southern » cluster is present in the southern part of the sampled area, corresponding to samples collected in Wyoming, Nebraska and Colorado. The « Mountain » cluster is observed in the western part of Montana and Idaho. The genetic structure of the North American *C*. *cinctus* populations revealed here is consistent with a previous study based on RAPD analysis [21]. As was observed in this study, three genetic clusters are present in the Northern Great Plains. However, their study was restricted to Montana, North Dakota and considered only one population from Wyoming. The sampling scheme applied here integrates for the first time, samples from the recent southern outbreaks as well as samples from Canada–where the species has been considered as a serious economic pest for a long time. The sampling scheme also includes more samples from western Montana and Idaho. This allows better identification of the extent and the distribution of the different genetic clusters. Contrary to Lou et al [21] who observed only two clusters in Montana, the mtDNA dataset suggests that three genetic clusters are present in Montana. This finding might be associated with biological properties of each cluster. The clear split into two different genetic clusters between eastern and western Montana can be linked to previous observations in adult emergence timing among sawflies from these two regions [21, 65]. Furthermore, a recent study including collection locations used here (*i*.*e*. Gallatin County for western Montana and Valley County plus Daniels County for eastern Montana) showed the same divergent pattern of the wheat stem sawfly development between western and eastern Montana [66]. Similar differences between northern *versus* southern populations were documented ([67, 68] and D. Cockrell unpublished data) and could also be due to genetic differences which is supported by the genetic clustering we found (Figs 1B and 4).

In phytophagous insects, host plant, habitat fragmentation, reproductive system, dispersal capacity and geographical or reproductive barrier are reported as the main drivers of genetic structure [16, 17, 69]. In this study, no conclusion can be drawn regarding host plant as only two genera (*i*.*e*. wheat and three *Bromus* spp. that are similar in appearance), which both include exotic species, are considered. Regarding the genetic pattern observed across the Rocky Mountains, this area corresponds to a less disturbed environment where the wheat cover is less abundant than in the others sampled localities. Rand et al [70] found consistent and significant increases in infestation levels of *C*. *cinctus* in response to increasing amounts of landscape-level wheat cover although no correlation was found between this variable and *C*. *cinctus* infestation, as might be expected. The Mountain cluster could be best explained by the topology of this area. The Rocky Mountains correspond to an area of rugged topology with isolated arable valleys that might act as physical barriers limiting the gene flow between populations and might explain the highest level of differentiation observed. Isolated populations with limited gene flow with others increase the chance of inbreeding as supported by relatively high positive *F*_IS_ values observed in several populations from the Mountain cluster.

High genetic diversity within populations is highly advantageous for colonizing species as it enables responses to selection pressure. Some wheat production practices such as the increase of no-till agriculture, provide a favorable environment for this insect. This practice may enhance the sawfly overwintering survival, increasing the population size and its potential range expansion [1, 23, 70]. It is not clear yet whether recent winter wheat infestations in the southern regions result from local populations evolving to exploit winter wheat or if it corresponds to the southerly range expansion of an adapted strain [4, 71]. The clustering of the southern populations with individuals collected in eastern Montana and North Dakota in the SAMOVA and the basal position of the population sampled in North Dakota in the NJ tree may suggest introductions from previously infested areas. However, during the initial stages of colonization of an organism, one expects evidence of bottlenecks such as a reduction of heterozygosity, a reduced allelic richness or genetic diversity [59, 60]. Therefore the high haplotype diversity, the allelic richness as well as the heterozygosity observed in the Southern populations combined with the clustering pattern tend to exclude the hypothesis of a recent introduction of the wheat stem sawfly from the northern part of the wheat production area. The most likely scenario seems to be the result of the adaptation of local populations to wheat from hosts in surrounding wildlands.

### Implications for pest management

Our results support the North American origin of *C*. *cinctus*. These findings are important for management, particularly when the use of biological control agents is considered [23, 61, 66]. The choice of natural enemies from the native area of the pest in biological control programs relies on parallel evolution or at most co-evolution between natural enemies and their hosts. Failure to identify the correct origin of a pest or its misidentification may cause the use of unsuitable species as biocontrol agents and may negate efforts towards an effective program [72]. The results presented here therefore support maximizing the efficacy of the North American parasitoids, such as *Bracon cephi* and *Bracon lissogaster* [2, 66], for the biological control of the wheat stem sawfly.

We observed a parallel pattern of the wheat stem sawfly genetic diversity for individuals collected in wheat and grasses. The three haplogroups have been detected both for wheat and grasses and only a low (and non-significant) fraction of the structure was attributed to host plants. However, the absence of an “host effect” remains tentative as the sampling on grasses was restricted to Montana (mainly distributed in western Montana) and, most of individuals were swept in sites where one genus of grass, actually three members of *Bromus* spp. were highly abundant. Species composition at these sites could consist of an exotic subspecies of *Bromus inermis* (*Bromus inermis* ssp. *inermis*), or smooth brome, introduced into North America in the late 1880’s and is commonly found in wheat fields or in close proximity [73, 74], and/or a native subspecies of *Bromus inermis* (*Bromus inermis* ssp. *pumpellianus*), or Pumpelly’s brome, and/or the native mountain brome, *Bromus marginatus*. Smooth brome, Pumpelly’s brome and mountain brome are found in all areas where *C*. *cinctus* samples were collected. Nevertheless, 16 haplotypes were found at sites where these congeneric populations were, therefore some members of this genus could act as a reservoir for the wheat stem sawfly. However, smooth brome would not be a good host in terms of suitability, despite its apparent attractiveness to foraging and ovipositing adults [75]. By 1928, smooth brome was recommended as a permanent trap around wheat fields, after three years of evaluation [76]. This assessment of the role of host plants will require confirmation through a more detailed sampling on different grasses in wildlands, specifically on native North American species and also via collection from both grasses and wheat in the same field.

The present study shows that there are three different genetic clusters distributed across the North American wheat production area. These distinct evolutionary units, with potentially different biological characteristics [21, 65, 66], may respond differently to control measures and therefore should be considered as different entities from a biological standpoint and different management units for any type of control.

Another interesting point highlighted by the results is the discrepancy between the assumed low mobility of the wheat stem sawfly [10] and the observed pattern. A weak but significant effect of isolation by distance was found within the whole dataset. However, Mantel tests for IBD within each cluster were non-significant. This indicates that gene flow is relatively spatially unrestricted within the identified clusters and that the IBD signal detected in the whole dataset is likely created by greater population subdivisions and is not an artifact generated by IBD. However, low mobility of this species would strongly limit the rate of gene flow between populations. Therefore, given the low differentiation observed with the microsatellite data between populations and the large scale distribution of the different clusters (particularly the Northern cluster), recent and long dispersal event(s) may have occurred, contrary to the expectations for a low mobility. The life span of adults is short (less than 7 days) but they can accidentally be transported from one place to another and direct transportation of living larvae of *C*. *cinctus* for long distances is unlikely, but not impossible [1]. However, it is important to note that several things have changed in the traditional *C*.*cinctus* habitat that is now dominated by wheat. Farms have become larger and often wheat fields for a single farming operation can be separated by more than 20 miles. Thus, necessary flights across fallow land can be a greater distance than in the past. Little is known about the dispersal ability of sawflies in general, however it is considered that they weakly disperse by active flights [77]. For the wheat stem sawfly, adults of both sexes are reported to be weak fliers and rarely fly long distances at one time [10]. Typically, the adults fly close to the soil substrate and travel by making a series of brief flights. This appears more pronounced than when first described a century ago, because traversing the larger fields of modern agriculture requires greater distances. Yet, given the ready availability of crop and wildland grasses, they typically still do not need to fly very far. Very seldom do they rise considerably above the crop canopy and make longer, more directed and purposeful flights (D. Weaver, pers. obs.).

Other practices that have changed in recent years relate to cultivation and landscape practices. Revegetation efforts in modern prairie landscaping and disturbed areas, including highways [78, 79], with native grasses is accomplished using domesticated cultivars of favored grass hosts of *C*. *cinctus*. Western wheatgrass, *Pascopyrum smithii* (Rydb.) A. Love, widely used in private and commercial settings, is one example of widespread planting of *C*. *cinctus* host plants in locations where they have been displaced for many years. It is not possible to determine if changes in wheat cultivation or increasing restoration of native hosts of *C*. *cinctus* are important in the rapid spread of new populations in the southern part of its range in winter wheat. However, movement of *C*. *cinctus* populations may be less impaired with patchy localized restoration of hosts across urban and suburban landscapes and increased planting of favored hosts in transportation corridors.

A further implication of this finding is an overall high risk of spread of resistance alleles, due to the apparent gene flow, at least within clusters. One strategy used to control wheat stem sawfly populations is the development of new resistant cultivars, primarily solid-stemmed wheat varieties [1, 80, 81]. The emergence and the subsequent spread of well adapted populations to resistant cultivars, as observed with the western corn rootworm, *Diabrotica virgifera virgifera* [82], could potentially be promoted by the high genetic diversity found in the wheat stem sawfly.

## Supporting Information

S1 Appendix DNA protocols. (DOCX)

S1 Table Collection data for specimens identified based on their morphology and their respective GenBank accession numbers for mitochondrial sequences (*16S* and *COI*) obtained in this study. (DOCX)

S2 Table Sampling details and summary statistics of genetic diversity of the sampled North American *Cephus cinctus* populations used in this study. (DOCX)

S3 Table Pairwise *F*_ST_ estimates between the different sampled populations of *Cephus cinctus*. (DOCX)

S1 Fig Results of delineated genetic groupings identified by SAMOVA. Values of fixation index, *F*_CT_ for K = 2–10 groups.
(DOCX)
