# Supplementary material for: Phylogeography of the Wheat Stem Sawfly, Cephus cinctus Norton (Hymenoptera: Cephidae): Implications for Pest Management
Source: PLoS One. 2016 Dec 13;11(12):e0168370. doi: 10.1371/journal.pone.0168370 (PMC5154603; doi:10.1371/journal.pone.0168370)
Supplement: S1 Appendix — (DOCX) [file pone.0168370.s001.docx]

**Phylogeography of the Wheat Stem Sawfly, *Cephus cinctus* Norton (Hymenoptera: Cephidae): Implications for Pest Management**

Vincent Lesieur, Jean-François Martin, David K. Weaver, Kim A. Hoelmer, David R. Smith, Wendell L. Morrill, Nassera Kadiri, Franck B. Peairs, Darren M. Cockrell, Terri L. Randolph, Debra K. Waters and Marie-Claude Bon

**Insight on the Origin of the Wheat Stem Sawfly**

Polymerase chain reaction (PCR) amplifications were conducted for two mitochondrial gene fragments: a 264-bp region of the cytochrome oxidase I (COI) and 442 bp of the ribosomal 16S RNA (16S).

The COI mitochondrial region was partially amplified with C1-J-2797 (5’‑CCTCGACGTTATTCAGATTACC‑3’) and TL2-N-3014 (5’‑TCCAATGCACTAATCTGCCATATTA‑3’) pair of primers as described by Simon et al. [1]. The PCR reaction mix contained, in a final volume of 30 µL, 1X Qiagen buffer, 0.2 mM of each dNTP, 0.3 µM of each primer, 2 U of Taq polymerase, 1.5 mM of MgCl_2_, 0.2 mg.mL^-1^ of BSA and 2 µL of genomic DNA diluted at 1/10. PCR were performed using the following programs : 94°C for 3 min, followed by 2 cycles of 94°C for 30 s, 48°C°C for 1 min and 72°C for 1.5 min, then 7 cycles of 94°C for 30 s, 49/55°C°C for 1 min and 72°C for 1.5 min followed by 31 cycles of 94°C for 30 s, 55°C°C for 1 min and 72°C for 1 min and finished by a final elongation at 72°C for 7 min. The purified PCR product was directly sequenced in both directions by Genoscreen (Lille, France) using an ABI PRISM 377 DNA sequencer.

A 442 bp fragment of mitochondrial 16SrRNA gene was amplified using the forward primer LR-J-13017: 5’‑TTACGCTGTTATCCTAA‑3’ [2] and the reverse primer LR-N-13398: 5’‑CGCCTGTTTAACAAAAACAT‑3’ published in Simon et al. [1]. The PCR reaction mix contained, in a final volume of 30 µL, 1X Qiagen buffer, 0.2 mM of each dNTP, 0.3 µM of each primer, 2 U of Taq polymerase, 1.5 mM of MgCl_2_, 0.2 mg.mL^-1^ of BSA and 2 µL of genomic DNA diluted at 1/10. PCR were performed using the following programs : 94°C for 3 min, followed by 2 cycles of 94°C for 30 s, 46°C°C for 1 min and 72°C for 1.5 min, then 6 cycles of 94°C for 30 s, 47/53°C°C for 1 min and 72°C for 1.5 min followed by 32 cycles of 94°C for 30 s, 55°C°C for 1 min and 72°C for 1 min and finished by a final elongation at 72°C for 7 min.

**Phylogeography of North American Wheat Stem Sawflies**

**Sequencing.** The COI mitochondrial region was partially amplified (762 bp) with C1-J-2183 (5’‑CAACATTTATTTTGATTTTTTGG‑3’) and TL2-N-3014 (5’‑TCCAATGCACTAATCTGCCATATTA‑3’) pair of primers as described by Simon et al. [1] (1994). The PCR reaction mix contained, in a final volume of 25 µL, 1X Qiagen buffer, 0.2 mM of each dNTP, 0.3 µM of each primer and 1 U of Taq polymerase and 2 µL of genomic DNA diluted at 1/10. PCR were performed using the following programs : 94°C for 3 min, followed by 35 cycles of 94°C for 30 s, 52/54°C°C for 30 s and 72°C for 1 min and finished by a final elongation at 72°C for 7 min or (for recalcitrant samples) 5 cycles of 94°C for 30 s, 48°C for 30 s and 72°C for 1 min , followed by 30 cycles of 94°C for 30 s, 52°C for 30 s and 72°C for 1 min, and finished by a final elongation at 72°C for 7 min. Sample amplifications were sequenced by GenoScreen as previously described.

**Genotyping.** The five microsatellite markers developed by Hartel et al. [3] were used in this study. Amplifications reactions were performed in a total volume of 10 µL containing 10ng of genomic DNA as template, 5 µL of Qiagen multiplex PCR buffer (5U *Taq*), 0.10 µL of both forward and reverse primers (except for SAWM40 for which 0.20 µL of both forward and reverse primers were used); the first of which was 5´-fluorescence-labelled with one of the following dyes 6-FAM, PET, VIC, NED (Life Technologies) and were carried out in a Perkin Elmer 9700 Thermocycler (Applied Biosystem). SAWM40 was used alone whereas the other markers were multiplexed. The PCR conditions were as follow: 95°C for 15 min, 35 cycles at 94°C for 30 s, 60°C for 60 s, 72°C for 60 s and a final elongation at 72°C for 10 min. Then, 2 µl of each of a 1/1100 to 1/1300 dilution of the pooled fluorescently-labeled PCR products were mixed with 6 µl of formamide, 11.9 µl molecular grade water and 0.1 µl of *GeneScan*™ - *500 LIZ*® internal size standard and analyzed on an ABI3500 capillary sequencer at Montpellier 2 University (France). Fragment size was determined using GENEMAPPER version 5.0 (Applied Biosystems).

**References**

1. Simon C, Frati F, Beckenbach A, Crespi B, Liu H, Flook P. Evolution, weighting, and phylogenetic utility of mitochondrial gene sequences and a compilation of conserved polymerase chain reaction primers. Annals of the entomological Society of America. 1994;87(6):651-701.

2. Kambhampati S, Smith PT. PCR primers for the amplification of four insect mitochondrial gene fragments. Insect Molecular Biology. 1995;4(4):233-6.

3. Hartel KD, Frederick BA, Shanower TG. Isolation and characterization of microsatellite loci in wheat stem sawfly *Cephus cinctus* and cross-species amplification in related species. Molecular Ecology Notes. 2003;3(1):85-7.
