# Supplementary material for: Phylogeography of the Wheat Stem Sawfly, Cephus cinctus Norton (Hymenoptera: Cephidae): Implications for Pest Management
Source: PLoS One. 2016 Dec 13;11(12):e0168370. doi: 10.1371/journal.pone.0168370 (PMC5154603; doi:10.1371/journal.pone.0168370)
Supplement: S1 Table — (DOCX) [file pone.0168370.s002.docx]

S1 Table. Collection data for specimens identified based on their morphology and their respective GenBank accession numbers for mitochondrial sequences (*16S* and *COI*) obtained in this study.

| Species | Locality | Lattitude | Longitude | Date | Sex | Codes | Genbank acesssion n° | |
| --- | --- | --- | --- | --- | --- | --- | --- | --- |
|  |  |  |  |  |  |  | *COI* | *16S* |
| *Cephus cinctus* | Laura, Saskatchewan - Canada | 51.839500 | -107.308400 | 2002 | ♀ | Cin_255 | KX880590 | KX880608 |
|  | Port Oungre, Saskatchewan - Canada | 49.017800 | -103.809800 | 2002 | ♀ | Cin_37 | KX880588 | KX880606 |
|  | Fort Peck, eastern Montana - USA | 47.633717 | -106.152967 | 2007 | ♂ | Cin_10 | KX880587 | KX880605 |
|  | Table Mountain, western Montana - USA | 46.060000 | -111.718600 | 2002 | ♀ | Cin_9w | KX880589 | KX880607 |
|  | Washington County, Colorado - USA | 40.342980 | -103.291900 | 2014 | ♀ | Cin_14 | KX880591 | KX880609 |
|  | Skalkaho, western Montana - USA | 46.193300 | -113.432100 | 2006 | ♀ | Cin_20d | KX880592 | KX880610 |
|  | W. Fork Rock Creek, western Montana - USA | 46.216433 | -113.853167 | 2007 | ♀ | Cin_4d | KX880593 | KX880611 |
|  |  |  |  |  |  |  |  |  |
| *Cephus fumipennis* | Lanzhou, Gansu Province - China | 36.06108 | 103.834300 | 2002 | ♂ | Fum_01 | KX880585 | KX880603 |
|  | Lanzhou, Gansu Province - China | 36.06108 | 103.834300 | 2002 | ♀ | Fum_02 | KX880583 | KX880601 |
|  | Lanzhou, Gansu Province - China | 36.06108 | 103.834300 | 2002 | ♀ | Fum_0d | KX880586 | KX880604 |
|  |  |  |  |  |  |  |  |  |
| *Cephus hyalinatus* | Shenbunino, Sakhalin Oblast - Russia | 46.433100 | 141.857733 | 1971 | ♀ | Hya_Shen | KX880577 | KX880595 |
|  | Yuzhno-Sakhalinsk city, Sakhalin Oblast - Russia | 46.919667 | 142.679783 | 1971 | ♂ | Hya_YuSa | KX880578 | KX880596 |
|  | Primorskoe village, Primorsky Krai - Russia | 43.065250 | 131.497917 | 1966 | ♂ | Hya_Prim | KX880579 | KX880597 |
|  | Malokuril'skaya, Shikotan Island - Russia | 43.867233 | 146.829650 | 1971 | ♂ | Hya_Kuri | KX880580 | KX880598 |
|  | Krabozavodsk, Shikotan Island - Russia | 43.824800 | 146.757467 | 1971 | ♀ | Hya_Krab | KX880581 | KX880599 |
|  | Alekhino, Kunashir Island - Russia | 44.057917 | 145.824283 | 1971 | ♂ | Hya_Alek | KX880582 | KX880600 |
|  | Halawubeigou, Inner Mongolia - China | 38.850017 | 105.850000 | 2002 | ♀ | Hya_InMo | KX880584 | KX880602 |
|  |  |  |  |  |  |  |  |  |
| *Cephus pygmaeus* | Solaklı, Adana - Turkey | 37.357333 | 35.069317 | 1999 | ♀ | Pyg_03 | KX880594 | KX880612 |
