# Supplementary material for: Phylogeography of the Wheat Stem Sawfly, Cephus cinctus Norton (Hymenoptera: Cephidae): Implications for Pest Management
Source: PLoS One. 2016 Dec 13;11(12):e0168370. doi: 10.1371/journal.pone.0168370 (PMC5154603; doi:10.1371/journal.pone.0168370)
Supplement: S2 Table — (DOCX) [file pone.0168370.s003.docx]

S2 Table. Sampling details and summary statistics of genetic diversity of the sampled North American *Cephus cinctus* populations used in this study.

| Country | Province / State | Code | Lattitude | Longitude | Host | Year | mtDNA | |  |  |  |  |  |  |  |  | microsatellites | | |  |  |
| --- | --- | --- | --- | --- | --- | --- | --- | --- | --- | --- | --- | --- | --- | --- | --- | --- | --- | --- | --- | --- | --- |
|  |  |  |  |  |  |  | *n* | H | Hd | ± SD | π | ± SD | Tajima's *D* | *p* | Fu's *FS* | *p* | *n* | *AR* | *Ho* | *He* | *Fis* |
| Canada | Alberta | 101WM | 50.431400 | -113.290600 | Wheat | 2002 | 7 | 2 | 0.286 | 0.196 | 0.000375 | 0.000512 | -1.006 | 0.220 | -0.095 | 0.217 | 18 | 6.202 | 0.585 | 0.639 | 0.085 |
|  |  | Leth | 49.720000 | -112.800000 | Wheat | 1998 | 1 | 1 | / | / | / | / | / | / | / | / |  |  |  |  |  |
|  |  | 105WM | 49.973000 | -111.167500 | Wheat | 2002 | 5 | 2 | 0.600 | 0.175 | 0.002362 | 0.001896 | 1.573 | 0.970 | 2.429 | 0.852 | 20 | 6.560 | 0.690 | 0.679 | -0.016 |
|  |  | 110WM | 49.633400 | -111.952000 | Wheat | 2002 | 2 | 1 | / | / | / | / | / | / | / | / |  |  |  |  |  |
|  |  | ManyB | 49.430000 | -110.680000 | Wheat | 2002 | 1 | 1 | / | / | / | / | / | / | / | / |  |  |  |  |  |
|  |  | 108WM | 51.448300 | -110.618300 | Wheat | 2002 | 4 | 2 | 0.500 | 0.265 | 0.001312 | 0.001300 | -0.710 | 0.271 | 1.099 | 0.618 |  |  |  |  |  |
|  | Saskatchewan | 234TS | 52.604300 | -109.145700 | Wheat | 2002 | 7 | 1 | 0.000 | 0.000 | 0.000000 | 0.000000 | 0.000 | 1.000 | 0.000 | NA | 20 | 6.802 | 0.610 | 0.661 | 0.077 |
|  |  | 237TS | 52.417900 | -107.299600 | Wheat | 2002 | 2 | 1 | 0.000 | 0.000 | 0.000000 | 0.000000 | 0.000 | 1.000 | 0.000 | NA |  |  |  |  |  |
|  |  | 232TS | 52.002500 | -109.145600 | Wheat | 2002 | 2 | 1 | / | / | / | / | / | / | / | / |  |  |  |  |  |
|  |  | 242TS | 52.143800 | -105.878800 | Wheat | 2002 | 2 | 1 | / | / | / | / | / | / | / | / |  |  |  |  |  |
|  |  | 255TS | 51.839500 | -107.308400 | Wheat | 2002 | 9 | 4 | 0.694 | 0.147 | 0.002187 | 0.001603 | -1.069 | 0.190 | 0.009 | 0.488 | 22 | 6.572 | 0.618 | 0.650 | 0.050 |
|  |  | 111WM | 50.901900 | -108.761400 | Wheat | 2002 | 7 | 2 | 0.286 | 0.196 | 0.000375 | 0.000512 | -1.006 | 0.241 | -0.095 | 0.254 | 12 | 6.221 | 0.550 | 0.617 | 0.109 |
|  |  | 260TS | 50.593700 | -107.809800 | Wheat | 2002 | 2 | 1 | / | / | / | / | / | / | / | / |  |  |  |  |  |
|  |  | 199TS | 51.129800 | -106.585600 | Wheat | 2002 | 2 | 1 | / | / | / | / | / | / | / | / |  |  |  |  |  |
|  |  | 076TS | 50.467550 | -106.440070 | Wheat | 2001 | 2 | 1 | 0.000 | 0.000 | 0.000000 | 0.000000 | 0.000 | 1.000 | 0.000 | NA |  |  |  |  |  |
|  |  | 217TS | 50.640700 | -103.692300 | Wheat | 2002 | 2 | 1 | 0.000 | 0.000 | 0.000000 | 0.000000 | 0.000 | 1.000 | 0.000 | NA |  |  |  |  |  |
|  |  | 196TS | 50.589400 | -105.626400 | Wheat | 2002 | 7 | 3 | 0.714 | 0.127 | 0.003750 | 0.002564 | 0.847 | 0.813 | 2.091 | 0.881 | 17 | 5.804 | 0.550 | 0.606 | 0.092 |
|  |  | 209TS | 49.695100 | -106.004400 | Wheat | 2002 | 7 | 4 | 0.714 | 0.181 | 0.002625 | 0.001919 | -0.099 | 0.464 | -0.132 | 0.406 | 20 | 6.549 | 0.640 | 0.641 | 0.001 |
|  |  | 206TS | 49.132300 | -105.548300 | Wheat | 2002 | 9 | 3 | 0.722 | 0.097 | 0.002698 | 0.001888 | 1.612 | 0.964 | 1.855 | 0.844 | 7 | 5.800 | 0.686 | 0.638 | -0.075 |
|  |  | 037TS | 49.017800 | -103.809800 | Wheat | 2002 | 7 | 1 | 0.000 | 0.000 | 0.000000 | 0.000000 | 0.000 | 1.000 | 0.000 | NA | 20 | 6.483 | 0.617 | 0.664 | 0.071 |
|  |  | 188TS | 49.960200 | -102.961700 | Wheat | 2002 | 9 | 1 | 0.000 | 0.000 | 0.000000 | 0.000000 | 0.000 | 1.000 | 0.000 | NA | 18 | 6.586 | 0.576 | 0.659 | 0.125 |
|  |  | 156TS | 49.191600 | -101.911600 | Wheat | 2002 | 4 | 1 | 0.000 | 0.000 | 0.000000 | 0.000000 | 0.000 | 1.000 | 0.000 | NA |  |  |  |  |  |
| USA | North Dakota | Baker | 48.170000 | -99.670000 | Wheat | 2002 | 5 | 3 | 0.700 | 0.218 | 0.001837 | 0.001563 | -0.175 | 0.470 | 0.061 | 0.310 |  |  |  |  |  |
|  |  | 269TS | 46.893931 | -103.923700 | Wheat | 2006 | 6 | 4 | 0.800 | 0.172 | 0.007087 | 0.004591 | 0.167 | 0.564 | 1.326 | 0.718 | 19 | 7.296 | 0.642 | 0.728 | 0.118 |
|  |  | 272TS | 46.742950 | -104.063470 | Wheat | 2006 | 2 | 1 | 0.000 | 0.000 | 0.000000 | 0.000000 | 0.000 | 1.000 | 0.000 | NA |  |  |  |  |  |
|  |  | 274TS | 46.318930 | -102.724370 | Wheat | 2006 | 1 | 1 | / | / | / | / | / | / | / | / |  |  |  |  |  |
|  | Montana | 10FY | 47.633717 | -106.152967 | W. grasses | 2006 | 6 | 5 | 0.933 | 0.122 | 0.009711 | 0.006114 | 0.347 | 0.647 | 0.318 | 0.481 |  |  |  |  |  |
|  |  | 109WM | 47.550000 | -105.563000 | Wheat | 2002 | 4 | 2 | 0.667 | 0.204 | 0.010499 | 0.007389 | 2.241 | 0.995 | 5.100 | 0.983 |  |  |  |  |  |
|  |  | 102WM | 48.555300 | -110.022000 | Wheat | 2002 | 7 | 3 | 0.667 | 0.160 | 0.002625 | 0.001919 | 1.076 | 0.880 | 1.321 | 0.765 | 20 | 7.001 | 0.630 | 0.692 | 0.090 |
|  |  | 103WM | 48.574700 | -107.891100 | Wheat | 2002 | 2 | 1 | 0.000 | 0.000 | 0.000000 | 0.000000 | 0.000 | 1.000 | 0.000 | NA |  |  |  |  |  |
|  |  | 03TS | 48.838350 | -106.328120 | Wheat | 2001 | 7 | 3 | 0.667 | 0.160 | 0.002875 | 0.002063 | 1.633 | 0.942 | 1.508 | 0.794 | 19 | 6.582 | 0.611 | 0.632 | 0.034 |
|  |  | 100WM | 47.733000 | -111.245300 | Wheat | 2002 | 7 | 3 | 0.667 | 0.160 | 0.001000 | 0.000946 | -0.275 | 0.377 | -0.438 | 0.166 | 20 | 7.115 | 0.720 | 0.694 | -0.038 |
|  |  | Chester | 48.520000 | -110.970000 | Wheat | 1996 | 1 | 1 | / | / | / | / | / | / | / | / |  |  |  |  |  |
|  |  | Pendroy | 48.070000 | -112.330000 | Wheat | 2001 | 1 | 1 | / | / | / | / | / | / | / | / |  |  |  |  |  |
|  |  | Brady | 48.020000 | -111.830000 | Wheat | 2001 | 1 | 1 | / | / | / | / | / | / | / | / |  |  |  |  |  |
|  |  | 16DW | 46.995250 | -112.356283 | W. grasses | 2006 | 11 | 6 | 0.727 | 0.144 | 0.003818 | 0.002445 | -2.084 | 0.000 | -0.609 | 0.351 | 9 | 7.213 | 0.500 | 0.726* | 0.311 |
|  |  | CCMgA | 47.140183 | -110.792500 | W. grasses | 2006 | 9 | 5 | 0.806 | 0.120 | 0.004666 | 0.002967 | -1.225 | 0.106 | 0.423 | 0.576 | 10 | 5.584 | 0.680 | 0.646 | -0.053 |
|  |  | 19FY | 47.077050 | -109.882567 | W. grasses | 2007 | 5 | 3 | 0.800 | 0.164 | 0.003675 | 0.002713 | 1.124 | 0.821 | 1.220 | 0.747 | 10 | 6.849 | 0.600 | 0.720* | 0.167 |
|  |  | 1DW | 47.073600 | -109.279150 | W. grasses | 2007 | 5 | 1 | 0.000 | 0.000 | 0.000000 | 0.000000 | 0.000 | 1.000 | 0.000 | NA | 7 | 5.600 | 0.543 | 0.605 | 0.102 |
|  |  | CCMgB | 46.730950 | -110.851183 | W. grasses | 2006 | 21 | 5 | 0.748 | 0.052 | 0.008986 | 0.004919 | 1.661 | 0.963 | 5.899 | 0.974 | 22 | 7.082 | 0.682 | 0.718 | 0.050 |
|  |  | 11WM | 46.578900 | -109.735800 | Wheat | 2002 | 9 | 4 | 0.750 | 0.112 | 0.005322 | 0.003323 | -0.726 | 0.262 | 2.041 | 0.852 | 20 | 6.806 | 0.660 | 0.697 | 0.053 |
|  |  | 7WM | 46.430000 | -109.380000 | Wheat | 2002 | 4 | 2 | 0.500 | 0.265 | 0.001969 | 0.001757 | -0.754 | 0.238 | 1.716 | 0.753 |  |  |  |  |  |
|  |  | Molt | 45.870000 | -108.930000 | Wheat | 2002 | 8 | 3 | 0.607 | 0.164 | 0.002015 | 0.001530 | -0.020 | 0.449 | 1.018 | 0.690 | 10 | 6.683 | 0.560 | 0.672 | 0.167 |
|  |  | 10WM | 45.130000 | -109.270000 | Wheat | 1996 | 6 | 4 | 0.800 | 0.172 | 0.005074 | 0.003419 | -1.194 | 0.148 | 0.698 | 0.627 | 17 | 6.821 | 0.610 | 0.656 | 0.070 |
|  |  | 30FY | 45.419533 | -111.072033 | W. grasses | 2007 | 5 | 3 | 0.700 | 0.218 | 0.006824 | 0.004643 | -1.210 | 0.036 | 2.385 | 0.868 |  |  |  |  |  |
|  |  | 1WM | 45.830000 | -111.530000 | Wheat | 2001 | 6 | 4 | 0.800 | 0.172 | 0.009186 | 0.005810 | -0.371 | 0.411 | 1.837 | 0.776 | 17 | 5.997 | 0.612 | 0.682 | 0.103 |
|  |  | 9WM | 46.060000 | -111.718600 | Wheat | 2002 | 9 | 5 | 0.806 | 0.120 | 0.005833 | 0.003599 | -0.940 | 0.203 | 0.930 | 0.683 | 17 | 6.278 | 0.612 | 0.691* | 0.115 |
|  |  | 28WM | 46.316433 | -110.803400 | Wheat | 2007 | 3 | 1 | 0.000 | 0.000 | 0.000000 | 0.000000 | 0.000 | 1.000 | 0.000 | NA | 4 |  |  |  |  |
|  |  | CCMgC | 46.402778 | -111.333333 | W. grasses | 2006 | 3 | 2 | 0.667 | 0.314 | 0.000875 | 0.001091 | 0.000 | 1.000 | 0.201 | 0.395 | 4 |  |  |  |  |
|  |  | 20DW | 46.193300 | -113.432100 | W. grasses | 2006 | 4 | 3 | 0.833 | 0.222 | 0.025153 | 0.016965 | 0.041 | 0.678 | 3.753 | 0.917 | 15 | 3.952 | 0.191 | 0.512* | 0.628 |
|  |  | 4DW | 46.216433 | -113.853167 | W. grasses | 2007 | 10 | 4 | 0.733 | 0.100 | 0.014348 | 0.008069 | -0.562 | 0.306 | 5.954 | 0.990 | 12 | 4.524 | 0.400 | 0.570* | 0.298 |
|  | Idaho | 2WM | 43.630000 | -111.750000 | Wheat | 2001 | 6 | 3 | 0.733 | 0.155 | 0.004024 | 0.002804 | 0.957 | 0.834 | 1.860 | 0.845 | 9 | 6.266 | 0.667 | 0.765 | 0.129 |
|  | Wyoming | 014TS | 41.972289 | -104.239231 | Wheat | 2001 | 3 | 2 | 0.667 | 0.314 | 0.000875 | 0.001091 | 0.000 | 1.000 | 0.201 | 0.385 | 20 | 7.513 | 0.700 | 0.722 | 0.030 |
|  |  | 070TS | 41.751731 | -104.070531 | Wheat | 2001 | 10 | 4 | 0.644 | 0.152 | 0.004141 | 0.002646 | -1.865 | 0.014 | 1.677 | 0.824 | 22 | 7.421 | 0.696 | 0.742 | 0.062 |
|  |  | 068TS | 41.419181 | -104.400500 | Wheat | 2001 | 7 | 4 | 0.714 | 0.181 | 0.005374 | 0.003486 | -1.250 | 0.110 | 1.296 | 0.731 | 10 | 7.302 | 0.760 | 0.739 | -0.029 |
|  | Nebraska | 020TS | 41.741431 | -103.544450 | Wheat | 2001 | 8 | 2 | 0.429 | 0.169 | 0.003375 | 0.002299 | 0.518 | 0.701 | 4.286 | 0.967 | 12 | 7.073 | 0.767 | 0.770 | 0.004 |
|  |  | 071TS | 41.560769 | -103.927681 | Wheat | 2001 | 7 | 5 | 0.857 | 0.137 | 0.008624 | 0.005315 | 0.405 | 0.671 | 0.825 | 0.604 | 11 | 7.042 | 0.655 | 0.720 | 0.091 |
|  |  | 022TS | 41.554650 | -103.404470 | Wheat | 2001 | 2 | 1 | 0.000 | 0.000 | 0.000000 | 0.000000 | 0.000 | 1.000 | 0.000 | NA |  |  |  |  |  |
|  | Colorado | Col13 | 40.605359 | -103.846223 | Wheat | 2013 | 12 | 2 | 0.167 | 0.134 | 0.000219 | 0.000355 | -1.140 | 0.165 | -0.476 | 0.129 | 20 | 7.017 | 0.630 | 0.721 | 0.126 |
|  |  | 14S54 | 40.342980 | -103.291900 | Wheat | 2012 | 13 | 5 | 0.538 | 0.161 | 0.003230 | 0.002097 | -2.196 | 0.003 | 0.447 | 0.606 | 9 | 6.100 | 0.533 | 0.724* | 0.263 |
|  |  | 14S3 | 39.461390 | -103.445480 | Wheat | 2014 | 7 | 4 | 0.714 | 0.181 | 0.001125 | 0.001025 | -1.358 | 0.081 | -1.798 | 0.021 |  |  |  |  |  |
|  |  | 13S52 | 39.363390 | -102.091190 | Wheat | 2013 | 3 | 2 | 0.667 | 0.314 | 0.000875 | 0.001091 | 0.000 | 1.000 | 0.201 | 0.376 |  |  |  |  |  |
|  |  | 13S37 | 38.729280 | -102.665400 | Wheat | 2013 | 3 | 1 | 0.000 | 0.000 | 0.000000 | 0.000000 | 0.000 | 1.000 | 0.000 | NA |  |  |  |  |  |

For mtDNA: *n*: Number of individuals analysed; H: Number of haplotypes; Hd: Gene diversity and its standard deviation; r: Allelic richness after rarefaction; π: Nucleotide diversity and its standard deviation; Tajima's *D*  and Fu's *FS*: results of neutrality tests and their respective *p* – values. For microsatellites: *n*: Number of individuals analysed; AR: Allelic richness after rarefaction; Ho: observed heterozygosity; He: expected heterozygosity. * indicate significant deviation from HW equilibrium.; *Fis*: inbreeding coefficient ; W. grasses: wildland grasses.
