# Supplementary material for: Phylogeography of the Wheat Stem Sawfly, Cephus cinctus Norton (Hymenoptera: Cephidae): Implications for Pest Management
Source: PLoS One. 2016 Dec 13;11(12):e0168370. doi: 10.1371/journal.pone.0168370 (PMC5154603; doi:10.1371/journal.pone.0168370)
Supplement: S3 Table — (DOCX) [file pone.0168370.s004.docx]

S3 Table. Pairwise *F*_ST_ estimates between the different sampled populations of *Cephus cinctus*.

|  | *n* | Northern cluster | | | | | | | | | | | | | | | | | | | Mountain cluster | | | | | | | | | Southern cluster | | | | | | | |
| --- | --- | --- | --- | --- | --- | --- | --- | --- | --- | --- | --- | --- | --- | --- | --- | --- | --- | --- | --- | --- | --- | --- | --- | --- | --- | --- | --- | --- | --- | --- | --- | --- | --- | --- | --- | --- | --- |
|  |  | 101WM | 105WM | 234TS | 255TS | 111WM | 196TS | 188TS | 209TS | 206TS | 037TS | 03TS | 102WM | 100WM | CCMgA | 1DW | 19FY | 11WM | Molt | 10WM | 16DW | 28WM | CCMgB | CCMgC | 9WM | 1WM | 20DW | 4DW | 2WM | 268TS | 14TS | 16TS | 20TS | 71TS | 68TS | 14S54 | Col |
| 101WM | 18 | - |  |  |  |  |  |  |  |  |  |  |  |  |  |  |  |  |  |  |  |  |  |  |  |  |  |  |  |  |  |  |  |  |  |  |  |
| 105WM | 20 | 0,036 | - |  |  |  |  |  |  |  |  |  |  |  |  |  |  |  |  |  |  |  |  |  |  |  |  |  |  |  |  |  |  |  |  |  |  |
| 234TS | 20 | 0,010 | 0,024 | - |  |  |  |  |  |  |  |  |  |  |  |  |  |  |  |  |  |  |  |  |  |  |  |  |  |  |  |  |  |  |  |  |  |
| 255TS | 22 | 0,026 | 0,025 | **0,009** | - |  |  |  |  |  |  |  |  |  |  |  |  |  |  |  |  |  |  |  |  |  |  |  |  |  |  |  |  |  |  |  |  |
| 111WM | 12 | **0,006** | 0,035 | **-0,006** | **-0,003** | - |  |  |  |  |  |  |  |  |  |  |  |  |  |  |  |  |  |  |  |  |  |  |  |  |  |  |  |  |  |  |  |
| 196TS | 17 | 0,080 | 0,076 | 0,049 | 0,065 | 0,059 | - |  |  |  |  |  |  |  |  |  |  |  |  |  |  |  |  |  |  |  |  |  |  |  |  |  |  |  |  |  |  |
| 188TS | 18 | 0,024 | 0,037 | **0,005** | 0,021 | **0,020** | 0,035 | - |  |  |  |  |  |  |  |  |  |  |  |  |  |  |  |  |  |  |  |  |  |  |  |  |  |  |  |  |  |
| 209TS | 20 | 0,032 | 0,030 | 0,019 | **0,001** | **0,013** | 0,062 | 0,017 | - |  |  |  |  |  |  |  |  |  |  |  |  |  |  |  |  |  |  |  |  |  |  |  |  |  |  |  |  |
| 206TS | 7 | 0,083 | 0,053 | 0,058 | **0,022** | 0,084 | 0,117 | 0,066 | 0,027 | - |  |  |  |  |  |  |  |  |  |  |  |  |  |  |  |  |  |  |  |  |  |  |  |  |  |  |  |
| 037TS | 20 | 0,035 | 0,040 | 0,016 | 0,026 | **0,008** | 0,031 | **0,009** | 0,021 | 0,064 | - |  |  |  |  |  |  |  |  |  |  |  |  |  |  |  |  |  |  |  |  |  |  |  |  |  |  |
| 03TS | 19 | 0,039 | 0,035 | 0,019 | **0,000** | **0,015** | 0,073 | 0,033 | **0,005** | 0,029 | 0,036 | - |  |  |  |  |  |  |  |  |  |  |  |  |  |  |  |  |  |  |  |  |  |  |  |  |  |
| 102WM | 20 | 0,016 | **0,006** | **0,010** | 0,011 | 0,017 | 0,065 | 0,025 | 0,020 | 0,027 | 0,025 | 0,017 | - |  |  |  |  |  |  |  |  |  |  |  |  |  |  |  |  |  |  |  |  |  |  |  |  |
| 100WM | 20 | 0,023 | 0,011 | 0,015 | 0,017 | 0,017 | 0,067 | 0,022 | 0,024 | 0,044 | 0,026 | 0,028 | **-0,009** | - |  |  |  |  |  |  |  |  |  |  |  |  |  |  |  |  |  |  |  |  |  |  |  |
| CCMgA | 10 | 0,057 | 0,037 | 0,038 | 0,030 | 0,054 | 0,119 | 0,060 | 0,035 | 0,050 | 0,053 | 0,060 | 0,024 | 0,029 | - |  |  |  |  |  |  |  |  |  |  |  |  |  |  |  |  |  |  |  |  |  |  |
| 1DW | 7 | **0,032** | 0,027 | **0,011** | **0,019** | **0,004** | 0,076 | 0,031 | 0,028 | 0,078 | 0,045 | 0,026 | **0,018** | 0,028 | 0,064 | - |  |  |  |  |  |  |  |  |  |  |  |  |  |  |  |  |  |  |  |  |  |
| 19FY | 10 | **0,016** | 0,028 | **0,008** | 0,026 | 0,026 | 0,072 | 0,022 | 0,033 | 0,067 | 0,027 | 0,035 | **0,008** | **0,012** | 0,045 | 0,038 | - |  |  |  |  |  |  |  |  |  |  |  |  |  |  |  |  |  |  |  |  |
| 11WM | 20 | 0,035 | 0,021 | 0,014 | 0,025 | 0,029 | 0,065 | 0,029 | 0,037 | 0,055 | 0,032 | 0,037 | **0,009** | **0,009** | 0,043 | **0,027** | **0,012** | - |  |  |  |  |  |  |  |  |  |  |  |  |  |  |  |  |  |  |  |
| Molt | 10 | 0,025 | 0,035 | **0,019** | **0,013** | **0,012** | 0,049 | 0,023 | 0,026 | 0,060 | **0,016** | 0,021 | **0,018** | 0,014 | 0,070 | **0,027** | **0,014** | **0,011** | - |  |  |  |  |  |  |  |  |  |  |  |  |  |  |  |  |  |  |
| 10WM | 17 | 0,020 | 0,025 | **0,008** | **0,012** | **0,002** | 0,061 | 0,021 | 0,019 | 0,060 | 0,023 | 0,013 | **0,013** | 0,020 | 0,056 | **0,002** | **0,015** | **0,011** | **0,004** | - |  |  |  |  |  |  |  |  |  |  |  |  |  |  |  |  |  |
| 16DW | 9 | 0,055 | 0,055 | 0,049 | 0,057 | 0,082 | 0,107 | 0,060 | 0,064 | 0,084 | 0,058 | 0,073 | 0,045 | 0,041 | 0,070 | 0,086 | **0,029** | 0,047 | 0,050 | 0,064 | - |  |  |  |  |  |  |  |  |  |  |  |  |  |  |  |  |
| 28WM | 4 | 0,049 | 0,030 | 0,052 | 0,058 | 0,092 | 0,108 | 0,071 | 0,075 | 0,075 | 0,062 | 0,081 | **0,024** | 0,026 | 0,044 | 0,086 | **0,025** | **0,031** | **0,041** | 0,062 | **0,002** | - |  |  |  |  |  |  |  |  |  |  |  |  |  |  |  |
| CCMgB | 22 | 0,049 | 0,046 | 0,058 | 0,055 | 0,066 | 0,100 | 0,073 | 0,074 | 0,080 | 0,062 | 0,072 | 0,037 | 0,032 | 0,062 | 0,086 | 0,032 | 0,041 | 0,037 | 0,062 | 0,023 | **-0,018** | - |  |  |  |  |  |  |  |  |  |  |  |  |  |  |
| CCMgC | 4 | 0,052 | **0,016** | **0,029** | **0,033** | **0,047** | 0,097 | 0,051 | 0,045 | 0,075 | **0,041** | 0,039 | **0,028** | 0,024 | 0,055 | **0,042** | **0,019** | **0,023** | **0,019** | **0,031** | **0,017** | **-0,018** | **0,007** | - |  |  |  |  |  |  |  |  |  |  |  |  |  |
| 9WM | 17 | 0,084 | 0,081 | 0,101 | 0,105 | 0,114 | 0,145 | 0,109 | 0,115 | 0,127 | 0,104 | 0,123 | 0,082 | 0,073 | 0,114 | 0,136 | 0,060 | 0,080 | 0,076 | 0,101 | 0,037 | **-0,004** | **0,013** | **0,026** | - |  |  |  |  |  |  |  |  |  |  |  |  |
| 1WM | 17 | 0,055 | 0,062 | 0,067 | 0,073 | 0,093 | 0,121 | 0,081 | 0,085 | 0,103 | 0,079 | 0,091 | 0,053 | 0,052 | 0,071 | 0,110 | 0,035 | 0,056 | 0,059 | 0,081 | **0,023** | **-0,021** | **0,002** | **0,023** | **0,010** | - |  |  |  |  |  |  |  |  |  |  |  |
| 20DW | 15 | 0,126 | 0,123 | 0,114 | 0,127 | 0,132 | 0,168 | 0,132 | 0,118 | 0,191 | 0,129 | 0,133 | 0,105 | 0,125 | 0,183 | 0,136 | 0,112 | 0,132 | 0,116 | 0,105 | 0,166 | **0,154** | 0,140 | **0,145** | 0,164 | 0,137 | - |  |  |  |  |  |  |  |  |  |  |
| 4DW | 12 | 0,055 | 0,093 | 0,082 | 0,100 | 0,111 | 0,146 | 0,101 | 0,101 | 0,155 | 0,106 | 0,106 | 0,081 | 0,087 | 0,124 | 0,118 | 0,078 | 0,102 | 0,095 | 0,092 | 0,048 | **0,045** | 0,080 | **0,082** | 0,091 | 0,068 | 0,172 | - |  |  |  |  |  |  |  |  |  |
| 2WM | 9 | 0,109 | 0,073 | 0,101 | 0,112 | 0,139 | 0,169 | 0,122 | 0,127 | 0,110 | 0,121 | 0,124 | 0,081 | 0,078 | 0,103 | 0,143 | 0,078 | 0,095 | 0,102 | 0,121 | 0,067 | **0,022** | 0,055 | **0,009** | 0,054 | 0,065 | 0,214 | 0,142 | - |  |  |  |  |  |  |  |  |
| 268TS | 19 | 0,073 | 0,038 | 0,048 | 0,051 | 0,068 | 0,087 | 0,052 | 0,061 | 0,063 | 0,046 | 0,055 | 0,037 | 0,036 | 0,077 | 0,060 | 0,031 | 0,017 | 0,032 | 0,037 | 0,047 | **0,028** | 0,041 | **0,019** | 0,066 | 0,059 | 0,147 | 0,118 | 0,083 | - |  |  |  |  |  |  |  |
| 14TS | 20 | 0,064 | 0,041 | 0,052 | 0,044 | 0,068 | 0,091 | 0,057 | 0,061 | 0,051 | 0,059 | 0,058 | 0,032 | 0,029 | 0,063 | 0,062 | 0,034 | 0,020 | 0,035 | 0,051 | 0,046 | **0,013** | 0,031 | **0,028** | 0,057 | 0,045 | 0,146 | 0,119 | 0,073 | 0,012 | - |  |  |  |  |  |  |
| 16TS | 22 | 0,060 | 0,047 | 0,038 | 0,049 | 0,057 | 0,070 | 0,041 | 0,054 | 0,064 | 0,033 | 0,060 | 0,037 | 0,034 | 0,053 | 0,063 | 0,028 | 0,030 | 0,034 | 0,041 | 0,055 | 0,029 | 0,044 | 0,039 | 0,080 | 0,067 | 0,154 | 0,121 | 0,080 | 0,022 | 0,026 | - |  |  |  |  |  |
| 20TS | 12 | 0,073 | 0,058 | 0,051 | 0,063 | 0,056 | 0,109 | 0,066 | 0,073 | 0,094 | 0,055 | 0,070 | 0,050 | 0,046 | 0,072 | 0,068 | 0,032 | 0,044 | 0,057 | 0,056 | 0,065 | 0,057 | 0,064 | 0,055 | 0,096 | 0,083 | 0,182 | 0,136 | 0,079 | 0,032 | 0,039 | 0,017 | - |  |  |  |  |
| 71TS | 11 | 0,066 | 0,047 | 0,047 | 0,050 | 0,064 | 0,093 | 0,054 | 0,060 | 0,061 | 0,045 | 0,056 | 0,031 | 0,023 | 0,061 | 0,071 | 0,032 | 0,028 | 0,036 | 0,046 | 0,051 | **0,025** | 0,037 | **0,039** | 0,070 | 0,058 | 0,172 | 0,131 | 0,065 | 0,022 | **0,004** | **0,016** | 0,025 | - |  |  |  |
| 68TS | 10 | 0,083 | 0,049 | 0,065 | 0,058 | 0,090 | 0,110 | 0,076 | 0,073 | 0,065 | 0,075 | 0,060 | 0,037 | 0,037 | 0,072 | 0,092 | 0,035 | 0,028 | 0,050 | 0,058 | 0,056 | **0,019** | 0,032 | **0,031** | 0,058 | 0,056 | 0,176 | 0,132 | 0,066 | 0,023 | **0,003** | 0,033 | 0,038 | **0,007** | - |  |  |
| 14S54 | 9 | 0,136 | 0,116 | 0,112 | 0,121 | 0,142 | 0,132 | 0,109 | 0,132 | 0,117 | 0,104 | 0,127 | 0,099 | 0,098 | 0,123 | 0,151 | 0,069 | 0,078 | 0,094 | 0,104 | 0,098 | 0,067 | 0,085 | 0,092 | 0,115 | 0,105 | 0,221 | 0,189 | 0,111 | 0,052 | 0,050 | 0,028 | 0,053 | 0,056 | 0,057 | - |  |
| Col | 20 | 0,051 | 0,034 | 0,028 | 0,044 | 0,051 | 0,059 | 0,025 | 0,052 | 0,060 | 0,028 | 0,046 | 0,025 | 0,026 | 0,056 | 0,052 | 0,023 | 0,021 | 0,029 | 0,031 | 0,050 | **0,032** | 0,054 | **0,032** | 0,091 | 0,070 | 0,159 | 0,113 | 0,076 | **0,014** | 0,023 | **0,003** | 0,020 | **0,010** | 0,033 | **0,033** | - |

Non-significant pairwise differentiation tests are shown in bold typeface and grey background.
