# Supplementary material for: Phylogeography of the Wheat Stem Sawfly, Cephus cinctus Norton (Hymenoptera: Cephidae): Implications for Pest Management
Source: PLoS One. 2016 Dec 13;11(12):e0168370. doi: 10.1371/journal.pone.0168370 (PMC5154603; doi:10.1371/journal.pone.0168370)
Supplement: S1 Fig — Values of fixation index, FCT for K = 2–10 groups. (DOCX) [file pone.0168370.s005.docx]

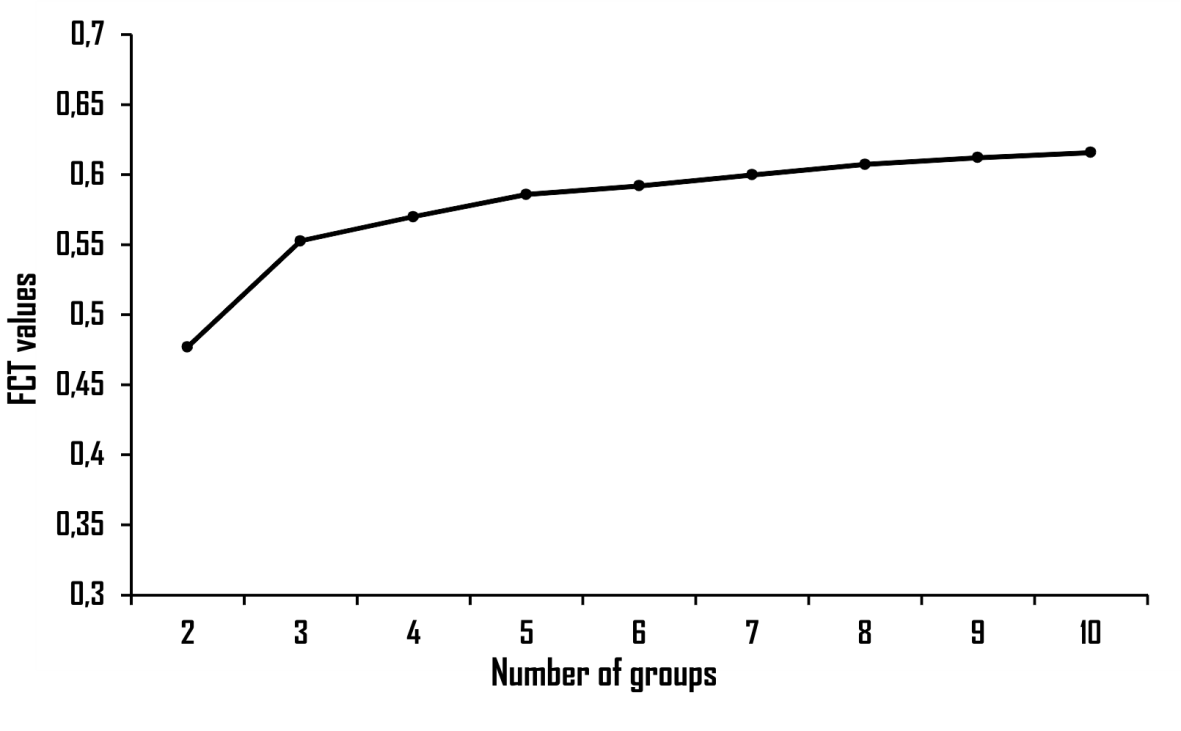


S1 Fig. Results of delineated genetic groupings identified by SAMOVA. Values of fixation index, *F*_CT_ for K = 2–10 groups.
